# Supplementary figures and images for: Interaction between Microsatellite Instability (MSI) and Tumor DNA Methylation in the Pathogenesis of Colorectal Carcinoma
Source: Cancers (Basel). 2021 Oct 1;13(19):4956. doi: 10.3390/cancers13194956 (PMC8508563; doi:10.3390/cancers13194956)

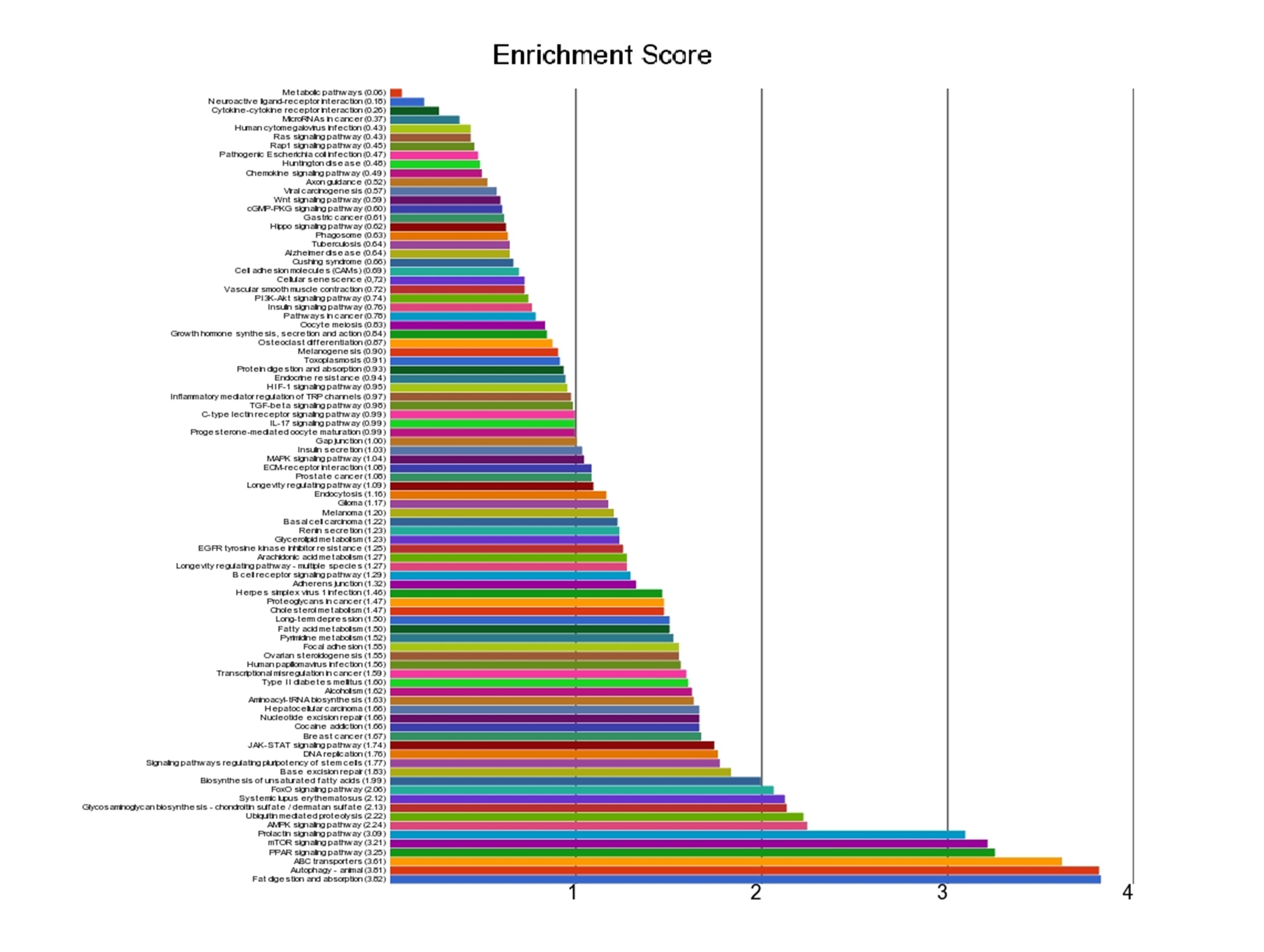

Supplement: Supplementary file 1 [file cancers-13-04956-s001.zip › Figure_S1.JPG]

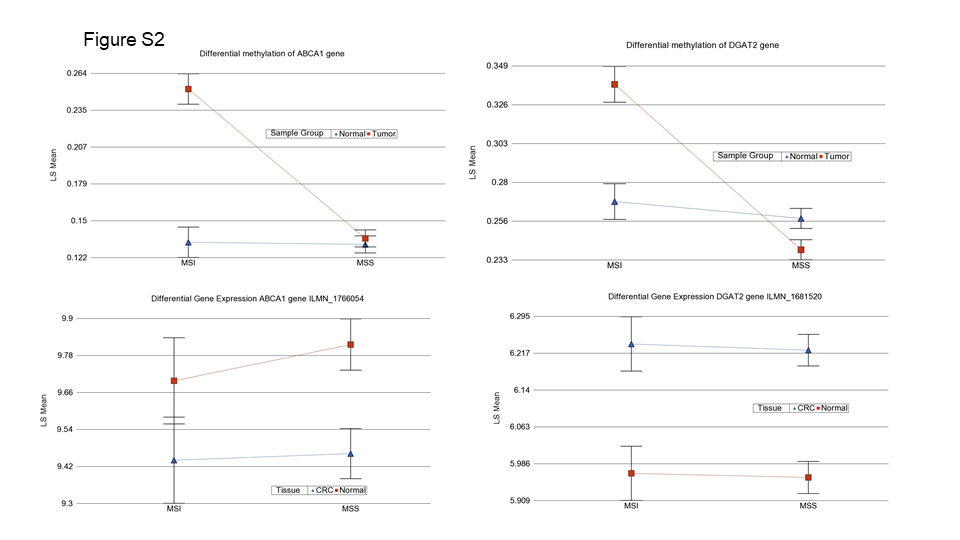

Supplement: Supplementary file 1 [file cancers-13-04956-s001.zip › Figure_S2.tif]
